# Effect of Low-Temperature Plasma on Porcine Oocytes In Vitro Primary Culture

**DOI:** 10.3390/antiox15050609

**Published:** 2026-05-12

**Authors:** Yuhan Wang, Panpan Guo, Haoyu Fang, Tingting Lu, Wencheng Song

**Affiliations:** 1 Anhui Province Key Laboratory of Medical Physics and Technology, Institute of Health and Medical Technology, Hefei Institutes of Physical Science, Chinese Academy of Sciences, Hefei 230031, China; 2 College of Biomedical Engineering, Anhui Medical University, Hefei 230032, China; 3 Key Laboratory for the Application and Transformation of Traditional Chinese Medicine in the Prevention and Treatment of Major Pulmonary Diseases, Anhui University of Chinese Medicine, Hefei 230012, China; 4 Collaborative Innovation Center of Radiation Medicine, Jiangsu Higher Education Institutions, School for Radiological and Interdisciplinary Sciences, Soochow University, Suzhou 215123, China

**Keywords:** porcine oocytes, low-temperature plasma, reactive oxygen species, primary culture

## Abstract

The effective growth and maturation of porcine oocytes in vitro are critical for advancing reproductive biotechnologies. In this study, we explored low-temperature plasma (LTP) treatment as a redox modulation strategy to enhance the survival and maturation of denuded porcine oocytes during in vitro primary culture in order to improve animal cellular health through innovative interventions. Freshly isolated oocytes were exposed to plasma treatment for different lengths of time and subsequently cultured under established in vitro conditions. Morphological and redox-related analyses showed that LTP treatment was associated with increased oocyte diameter, a higher first polar body extrusion rate, mitochondrial membrane potential changes, and altered intracellular and extracellular redox-related parameters. These beneficial effects exhibited a distinct time-dependent dose–response pattern. Furthermore, Western blot analysis showed altered expression of the EGFR/ERK signaling cascade and proteins such as Nrf2, suggesting that LTP treatment might participate in the regulation of maturation-related responses in porcine oocytes cultured in vitro by inducing redox-associated changes, along with alterations in EGFR/ERK-related signaling, Nrf2 expression, and molecules involved in maturation and apoptosis. Collectively, these findings highlight the positive role of LTP in supporting porcine oocyte maturation during in vitro primary culture and provide a promising approach for optimizing the in vitro primary culture of porcine oocytes.

## 1. Introduction

Mammalian oocytes develop from ovarian follicles, which are at various stages of maturation. Preantral follicles, in particular, are plentiful and house oocytes with significant developmental potential, making them valuable for both fundamental research and practical applications [1]. An essential technique in modern reproductive biology is in vitro primary culture technology, which facilitates the procurement of developmentally competent oocytes through the meticulous in vitro primary culture manipulation of oocytes sourced from preantral follicles [2]. Due to the close physiological resemblance between pigs and humans, in vitro primary culture technology for porcine oocytes not only offers plentiful cellular resources for porcine embryogenesis and piglet production [3] but also plays a vital role in transgenic porcine development [4], improving breeds and conserving endangered varieties [5], as well as in the clinical investigation of human reproductive disorders [6]. Oocyte quality has a direct effect on their potential for subsequent development. During the maturation of oocytes, cumulus cells and granulosa cells are instrumental in regulating their quality [7,8]. Previous studies have verified that the absence of cumulus cells during oocyte maturation markedly compromises the quality of in vitro maturation [9]. Although granulosa and cumulus cells are important in oocyte maturation, denudation of oocytes is inevitable in multiple assisted reproduction cycles and experiments, such as germinal-vesicle-stage oocyte nuclear transfer and RNA interference fragment injection [10]. However, porcine denuded oocytes have a low survival rate and maturation rate in current in vitro primary culture conditions [11]. Therefore, the establishment of a stable and effective in vitro primary culture system specifically for denuded oocytes is of paramount importance.

Previous related research has indicated that denuded oocytes can complete meiotic maturation during in vitro primary culture. For example, successful experiments have been reported in mice [12], rats [13], sheep [14], and cattle [15], confirming both the feasibility and developmental potential of in vitro primary culture of denuded oocytes (DOs). Although various in vitro primary culture approaches have been explored for porcine DOs, these remain suboptimal and have considerable limitations. In order to improve the efficiency of porcine oocyte culture in vitro, supplementation in medium with compounds such as cysteine, ascorbic acid, and vitamins has been employed, and relevant experimental results have shown that these manipulations enhance the developmental potential of porcine DOs [8,16,17]. However, the maturation quantity and quality of oocytes are not regulated by a single chemical substance, making these methods both highly demanding and significantly influenced by other factors. This ultimately leads to limited experimental results. Other studies have attempted to improve the maturation quality of porcine denuded oocytes by incorporating surrounding cells to form a co-culture system. These cells can be cumulus cells, mural granulosa cells, and ovarian cortical cells [18,19]. However, these studies have all utilized cultured monolayer cells, which do not accurately represent the in vivo form of cells. Such methods are not only complex in operation but also alter the properties of cells after separate cultivation, leading to no substantial improvement in the quality of porcine denuded oocytes [20]. Furthermore, numerous investigations have shown that various effects can be achieved by including antioxidants like melatonin in the culture medium [21] or by manipulating the expression of essential maturation genes using molecular biology methods to promote maturation [22]. Though some progress has been made in the field, its underlying mechanisms are complex and cause side effects or indirectly affect oocyte maturation. Specifically, the long-term application of these methods could modify the metabolic and physiological conditions of oocytes, making these techniques less universally applicable. Thus, it is crucial to seek out novel research strategies.

Plasma, which has been recognized as the fourth state of matter, exists independently of the other states (solid, liquid, and gaseous). Low-temperature plasma (LTP) functions at temperatures that are either at or slightly above ambient conditions. In controlled temperatures, LTP thermally damages biological tissue little and avoids environmental contamination [23]. These beneficial traits have sparked considerable research interest within biomedical fields, especially regarding tissue repair, regenerative medicine, and reproductive biology. Increasing evidence indicates that LTP treatment promotes the proliferation of skin fibroblasts and increases the number of cells that advance to the S phase [24]. Researchers have found that LTP promoted L929 cell proliferation, enhanced Cyclin D1 expression, and facilitated transition from the G1 to the S phase, which are evidence of LTP accelerating cell cycle passage and supporting cell maturation [25]. In addition, LTP exerts antioxidant protection against ovarian granulosa cells in an inflammatory environment, thereby reducing apoptosis rates and promoting cell maturation [26]. Therefore, these verdicts highlight the capability of LTP to improve cell growth and maturation in in vitro primary culture systems, which may ultimately enhance reproductive outcomes. Nevertheless, conventional dielectric barrier discharge plasma generators predominantly utilize rigid insulating materials such as quartz or ceramics as their dielectric layers [27,28]. Due to the rigidity of such materials, the instrument’s versatility is limited, and its application range is greatly constrained. On the other hand, flexible plasma pads are a new experimental device which represents an appealing approach to extending the use of LTP in vitro primary culture systems and promotes a broader use of LTP in reproductive biotechnology [29].

In this study, we explore LTP treatment as a new method to improve the survival and maturation rates of immature oocytes obtained from porcine ovarian cortical preantral follicles. A flexible plasma pad was employed, with different plasma doses being achieved by modifying the duration of exposure. The effect of LTP on oocytes was comprehensively evaluated through morphological assessments, cell viability tests, investigations of intra- and extracellular bioactive substances, and examinations of mitochondrial dynamics. In addition, Western blot (WB) analyses were conducted to clarify the mechanisms by which LTP facilitates the in vitro development of oocytes.

## 2. Materials and Methods

### 2.1. Oocyte Collections

Fresh ovarian oocytes were collected from the ovaries of adolescent Zongyang black pigs in Zongyang, China. Immediately after slaughter, the ovaries were placed at 0 °C. They were treated using 75% ethanol for disinfection and then rinsed with phosphate-buffered saline (PBS) that included 1% penicillin/streptomycin (Sangon Biotech, Shanghai, China) at 37 °C. The mesentery and ligaments on the surface of the ovaries were then removed using ophthalmic scissors, and the ovarian cortical tissue was carefully dissected with a scalpel to a depth of approximately 2 mm, yielding ovarian cortical pieces measuring about 5 mm in length and width. The fragments of processed porcine ovarian cortex were immersed in a follicle separation medium. This medium consisted of DMEM (high-glucose DMEM, Sangon Biotech, Shanghai, China), enriched with 5% fetal bovine serum (FBS) from the same company, 1000 mol·m^−3^ HEPES, and 1% penicillin/streptomycin, and was pre-warmed to 37.5 °C in a 60 mm culture dish. Preantral follicles showing well-preserved structures and healthy morphology with diameters between 200 and 300 micrometers were chosen under an inverted microscope using a 1-milliliter syringe with a 21-gauge needle [30]. After retrieval from preantral follicles, oocytes were washed in pre-warmed PBS supplemented with 1% penicillin/streptomycin. The surrounding granulosa/cumulus cells were removed mechanically by repeated gentle pipetting using a fine glass pipette under microscopic observation until no adherent somatic cells remained around the zona pellucida. Only oocytes with an intact zona pellucida, homogeneous cytoplasm, and complete removal of surrounding somatic cells were selected as denuded oocytes for subsequent culture.

### 2.2. LTP Treatment

The Flexible Plasma Pads apparatus utilized in this experiment has been validated before [29]. This apparatus comprises four components: a high-voltage insulation layer, a high-voltage electrode, a dielectric layer, and a ground electrode. The high-voltage insulation and dielectric layers are constructed from polyimide film, while the high-voltage and ground electrodes are made of copper. The experiment confirmed that the apparatus exhibits satisfactory electrical safety. The operating frequency of the equipment was 6.83 kHz, with a peak voltage of 4.8 kV. The flexible plasma pad used in this study was based on a dielectric barrier discharge (DBD) configuration, consisting of a multilayer structure including a high-voltage electrode, a dielectric barrier, and a ground electrode, all encapsulated within insulating polyimide films. Importantly, both electrodes were fully embedded within the insulating layers and were not exposed to the external environment. Therefore, no direct contact occurred between the electrodes and the culture medium, effectively preventing potential metal ion release; the encapsulated design of the copper electrodes also minimized the possibility of trace metal contamination. During treatment, the plasma device was positioned horizontally above the culture dish at a fixed distance of 0.5 cm from the medium surface, without direct contact. Under these conditions, plasma interacted primarily with the gas–liquid interface, leading to the generation of reactive species in the culture medium, and subsequently influenced oocytes indirectly. In this study, the plasma source, culture medium, and oocytes formed a vertically aligned system, in which reactive species were generated at the interface and diffused into the medium before interacting with the oocytes. Prior to each treatment, fresh medium was replaced promptly to ensure that the cell culture medium remained adequately nutritious. Additionally, in the experiment, we established a gradient of treatment times to accurately observe the effects of LTP on oocytes. N-acetyl-L-cysteine (NAC), a scavenger of ROS, was purchased from Beyotime (S0077, Shanghai, China) and was used as a redox-modulating reagent at a final concentration of 10.0 mM·L^−1^. In NAC-treated groups, NAC was added before LTP treatment in the experiments shown in Section 3.7 and Section 3.8.

### 2.3. Denuded Oocyte In Vitro Primary Culture

For primary culture in vitro, we first took 96-well plates and added 100 μL of a 0.2% gelatin solution (Beyotime, Shanghai, China, ST1339) to each well. Subsequently, the plates were placed in a humidified incubator (Thermo Fisher Scientific, Waltham, MA, USA) at 38.5 °C with 5% CO_2_ for 30 min to ensure humidity saturation. After the incubation period, the gelatin solution in the well plate was aspirated. Meanwhile, the culture medium, consisting of hormone-containing TCM199 maturation medium (Thermo Fisher Scientific, Waltham, MA, USA, product NO. 31100035), which included 1% penicillin/streptomycin, 1% ITS-A Media Supplement (Beyotime, Shanghai, China, C0343), 10 IU·mL^−1^ chorionic gonadotrophin (Ningbo Second Hormone Factory, Ningbo, China), 10 IU·mL^−1^ serum gonadotrophin (Ningbo Second Hormone Factory), and 5% FBS, was introduced to the 96-well plate at 200 μL per well. Next, 50 μL of mineral oil (Beyotime, Shanghai, China, ST275) was overlaid on each well, and the setup was pre-equilibrated in a cell culture incubator at 38.5 °C, 5% CO_2_, and saturated humidity for 2 h. Isolated oocytes were individually picked up and transferred into each well of the 96-well plate (one oocyte per well) using an oocyte transfer pipette. Porcine oocytes were subjected to continuous in vitro primary culture, with the culture medium replaced every 2 d throughout the experiment. During culture, oocyte morphology, apparent diameter, and perivitelline space formation were recorded as descriptive morphometric features.

### 2.4. Oocyte Viability Assay

The assessment of oocyte viability was conducted using a well-documented and validated method [31,32], and this experiment involved the application of MTT, which was 3-(4,5-dimethylmidazol-2-yl)-2,5-diphenyltetrazolium bromide from Sigma-Aldrich in St. Louis, MO, USA. Oocytes treated under different treatment conditions were exposed to a 1.2 mM MTT solution for 4 h at the end of 12 d of in vitro primary culture. After the incubation, the oocytes were washed four times with PBS. Following that, the cells were placed into a 96-well plate, and 100 μL of dimethyl sulfoxide (DMSO, Sengong Biotech, Shanghai, China) was introduced into each well. In each well, the absorbance was recorded by a microplate reader with a 492 nm wavelength [29]. Each experimental group comprised 50 oocytes, and the experiment was replicated three times. The final value was calculated based on a specific equation that considered the absorbance readings from different groups in the experiment. To determine this ultimate value, we subtracted the absorbance of the blank group from the same data of each experimental group.

### 2.5. First Polar Body Extrusion

Oocytes from various experimental treatments were transferred to a 6-well plate in each group of 30 cells. Subsequently, the oocytes were immersed in a staining solution containing 10 μg/mL Hoechst (Beyotime, Shanghai, China, C1022) and stained in the dark for about 30 min. Following staining, the oocytes were cleaned three times with PBS to eliminate excess dye. The first polar body was observed using a fluorescence microscope (Olympus, Tokyo, Japan). Hoechst staining emits blue fluorescence under ultraviolet light, allowing the first polar body to be distinctly observed as a small blue, fluorescent spot, positioned either outside or near the oocyte. The rate of first polar body extrusion, defined as the percentage of oocytes that effectively released the first polar body, was documented.

### 2.6. Extracellular Reactive Species Detection

Following the retrieval of oocytes, they were carefully placed into a culture dish with a diameter of 35 mm, containing 2 mL of specialized culture medium. Then, the cells were treated with flexible plasma, making sure that the distance from the plasma pad to the surface of the culture medium was 0.5 cm. Afterwards, the treated cells were incubated with the culture medium for 8 h. After the incubation period, we measured the concentration of reactive oxygen species (ROS) and reactive nitrogen species (RNS) using a reactive oxygen species assay kit (Beyotime, Shanghai, China, S0038) and a nitric oxide (NO) assay kit (Beyotime, Shanghai, China, S0021S). The concentration of final reactive species was determined using standard curve data generated from the relevant absorbance recorded at 540 nm. Each experimental group consisted of about 30 cells, and each experiment was run three times for reliability.

### 2.7. Assessment of Oxidative Stress

After initial LTP treatment and then 8 h of in vitro primary culture, the measurement of intracellular reactive oxygen species and intracellular glutathione (GSH) was carried out. The ROS measuring assay kit containing the reagent of 2′,7-dichlorodihydrofluorescein diacetate (DCFH-DA) was purchased from KeyGEN BioTECH in Nanjing, China (S0033S). Then, oocytes were incubated for 30 min in dark conditions at about 38.5 °C in an atmosphere with 5% CO_2_. After incubation, PBS was used to wash the oocytes three times to eliminate excess reagents. In the next step, oocyte fluorescence was observed using an Olympus fluorescence microscope made in Tokyo, Japan. We also measured GSH levels through a staining method, where 10 μM of the reagent 4-chloromethyl-6,8-difluoro-7-hydroxycoumarin (CMF2HC), produced by the company of Thermo Fisher Scientific in Waltham, MA, USA, with the catalog number C12881, was applied. The staining method was the same as that of detecting ROS. Thus, cells were kept in the dark for 30 min under the same conditions of temperature and CO_2_ concentration. The oocytes were subjected to three washes with PBS after the incubation period to remove the unbound dye and then observed as quickly as possible with the Olympus microscope through fluorescence (Tokyo, Japan). A total of about 30 oocytes were selected for analysis from each experimental group, and the whole experimental protocol was performed 3 times for validation. The fluorescence intensity of the samples was quantified using ImageJ 1.54f software.

### 2.8. Mitochondrial Membrane Potential Assay

The oocytes’ mitochondrial membrane potential was appraised using a mitochondrial membrane potential assay kit (Solarbio, Beijing, China). The oocytes were placed in a JC-1 staining solution at 38.5 °C and incubated in the dark for 30 min following culture. After incubation, the oocytes were washed two times with the JC-1 staining buffer at 4 °C. The fluorescence microscope helped to assess the changing red and green fluorescence intensity, while the ImageJ software was used for the analysis of the fluorescence intensity. The relative intensity between red and green fluorescence serves as a metric for assessing the mitochondrial membrane potential. This analysis was conducted on three separate occasions.

### 2.9. Western Blot Analysis

Oocytes were collected following the maturation process and subsequently lysed on ice for a duration of 20 min using RIPA cell lysis buffer sourced from a company in Shanghai, China. The experimental group comprised 200 oocytes that had successfully completed the cultivation process. Once the lysates were collected, they were centrifuged to separate the cellular components. A BCA protein detection kit, provided by the same company, was used to determine protein concentration. Following this quantification, a Western blot analysis was performed. Gel electrophoresis was performed by preparing SDS-PAGE gel using a kit from Beyotime. Every lane of the gel was loaded with a suitable amount (10 µL) of protein sample. After electrophoresis, the protein was transferred to the nitrocellulose membrane. To prevent nonspecific binding, the membrane was blocked with a 5% skim milk solution before proceeding to the next step. The membrane was incubated with diluted primary antibody at a density of 1:1000 for over 12 h at 4 °C. Subsequently, a secondary antibody (horseradish peroxidase (HRP), Cell Signaling, Danvers, MA, USA, at a density of 2:10,000) was applied for more than one hour. A chemiluminescence kit from Thermo Fisher Scientific, Waltham, MA, USA, and a specific chemiluminescence gel imaging system from Tanon, Shanghai, China, were adopted in this study. Multiple primary antibodies, including anti-EGFR (catalog number A12303), anti-Ras (catalog number A4735), anti-ERK1/2 (catalog number A16686), anti-p-ERK1/2 (Catalog number AP0485), anti-Nrf2 (catalog number A1244), anti-GDF9 (Catalog number A2739), anti-Cyclin B1 (catalog number A22435) and anti-Beta-Actin (catalog number AC026) from ABclonal in Wuhan, China, and anti-cleaved Caspase-3 (catalog number 341034) from Zen-bioscience, Chengdu, China, were employed.

### 2.10. Statistical Analysis

The experimental results were reported by calculating the mean and standard deviation, with each trial performed independently on three distinct occasions. An analysis of the experimental data used one-way as well as two-way ANOVA, with statistically significant differences indicated by * *p* < 0.05, ** *p* < 0.01, and *** *p* < 0.001. When there was a considerable difference, Tukey’s multiple comparison test was performed for post hoc pairwise comparisons to see which ones differed from each other.

## 3. Results

### 3.1. Establishment of Primary Culture In Vitro

**Figure 1:**
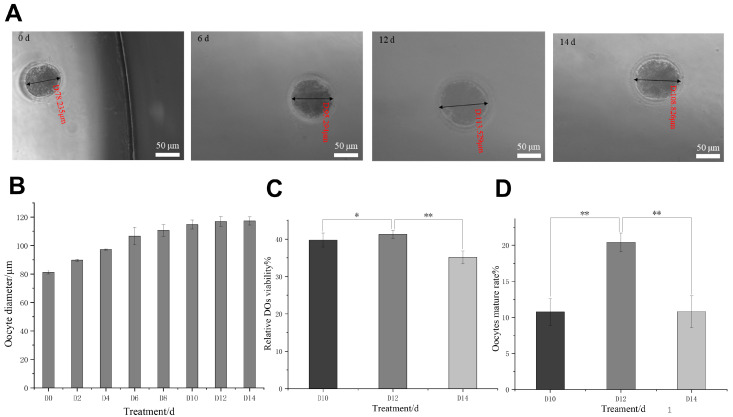
Primary culture in vitro. (**A**) Representative images of cells at 0 d, 6 d, 12 d, and 14 d of culture. Scale bar = 50 µm. (**B**) Changes in cell diameter during the culture period. (**C**) The survival rate of oocytes during the late culture period. (**D**) The mature rate of oocytes during the late culture period. The data indicated the mean ± SD from three separate investigations. *: *p* < 0.05 and **: *p* < 0.01; both were from ANOVA compared with the others.

To characterize the established culture system, the descriptive morphological changes in preantral-follicle-derived denuded oocytes were monitored throughout the culture period. As illustrated in Figure 1A, normally surviving oocytes display an intact morphology, characterized by a light and uniformly delicate cytoplasm devoid of noticeable granules or uneven shading. The cell contour is well-defined, and the shape is plump; the zona pellucida forms a complete, transparent ring around the cell, gradually thickening as oocytes progress through their growth period. Descriptive changes were observed in apparent oocyte diameter during the culture period: the cell diameter consistently increased, stabilizing after the first polar body was formed in the perivitelline space (PVS) (Figure 1B). In all groups, the survival rate of oocytes remained stable during the first 12 d of culture; however, it significantly decreased at 14 d compared to 12 d (Figure 1C). The cell diameter continued to increase during the first 12 d, although the rate of increase gradually slowed, peaking at 12 d, and then ceased to grow from 12 d to 14 d (Figure 1B). Notably, PVS had not yet formed during the initial stages of culture. However, as oocytes developed and matured, PVS gradually began to form and widen from 10 d, coinciding with the initial observation of first polar body formation within the PVS (Figure 1A). As illustrated in Figure 1D, the maturation rate of cells continued to rise from 10 d, peaking at 12 d, before declining at 14 d. Gradual changes in oocyte morphology and apparent diameter were observed during culture; these observations supported day 12 as the most suitable endpoint for subsequent experiments in this system.

### 3.2. Effect of LTP on Maturation of Oocytes

**Figure 2:**
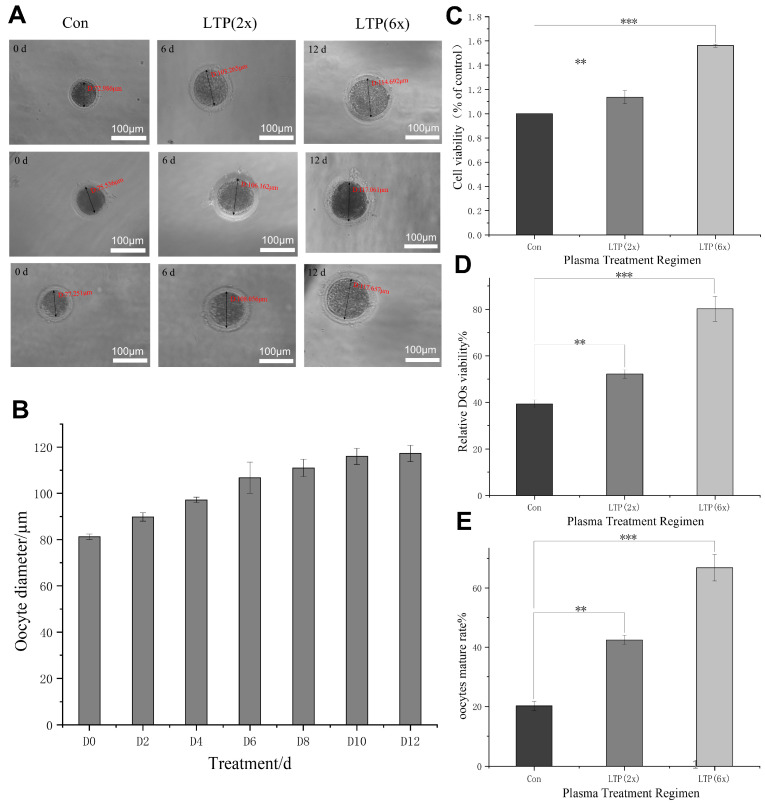
Effects of various treatments on oocytes. (**A**). Representative bright field images of control, LTP (2×), and LTP (6×) groups during the culture process are shown. Scale bar = 100 µm. (**B**). Changes in cell diameter in the LTP (2×) experimental group throughout the culture period are illustrated. (**C**). Cell viability across different experimental groups following the culture period. (**D**). The survival rates of cells from different experimental groups after the culture period. (**E**). The mature rate of oocytes in the various groups. The data is expressed as the mean ± standard deviation from three separate experiments. ** *p* < 0.01 and *** *p* < 0.001 according to ANOVA when compared to the other groups.

Oocytes freshly isolated from the ovarian cortex were subjected to direct treatment for 30 s using a flexible plasma pad, followed by cultivation. After 12 d of culture, we observed the cell diameters at 10 d and 12 d (Figure 2A,B). In addition to a single 30 s direct LTP treatment administered at the time of oocyte retrieval, we established indirect treatment groups: LTP (6×) denotes the use of medium pretreated with 30 s LTP exposure during each medium change throughout the culture period; LTP (2×) indicates that medium pretreated with 30 s LTP exposure was used for only one medium change during the culture period. By observing the morphology of oocytes in the control, LTP (2×), and LTP (6×) groups under a microscope, we found that cells in all three groups maintained normal growth until maturation (Figure 2A). Our findings indicate that the increase in cell diameter in the LTP (2×) experimental group stabilized prior to 12 d, which was earlier than in the control group (Figure 2B). Furthermore, by evaluating the cell survival percentage of the experimental group LTP (2×) at the conclusion of the culture period in relation to the control group, we observed an increase in the LTP (2×) experimental group, suggesting that LTP had positively influenced the success rate of the established cell culture system (Figure 2D). In juxtaposition to control, there was a notable rise in cell viability in both the LTP (2×) and LTP (6×) groups, with LTP (6×) showing a greater enhancement than LTP (2×) (Figure 2C). Upon analyzing the survival and maturation rates at the conclusion of the cell culture period, our findings suggested that these measurements were significantly higher in the experimental groups compared with the control and that the LTP (6×) group was superior to LTP (2×) (Figure 2D,E).

### 3.3. Optimization of LTP Time

**Figure 3:**
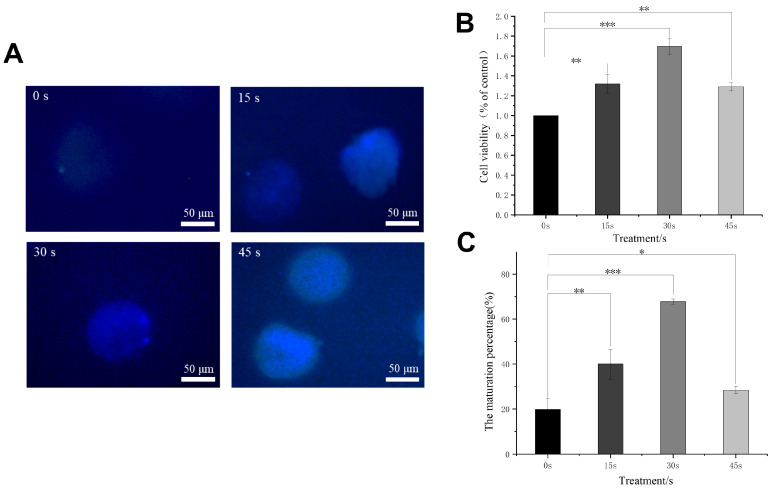
The effect of different durations of LTP treatment on the extrusion of the primary polar body of oocytes. (**A**). Representative fluorescence images showing nuclear maturation of oocytes, as indicated by Hoechst staining and first polar body extrusion. Scale bar = 50 µm. (**B**). Cell viability of different experimental groups after culture. (**C**). The frequency of first polar body extrusion across various experimental groups. The data show the mean ± SD derived from three separate verifications. * *p* < 0.05, ** *p* < 0.01, and *** *p* < 0.001, as determined by ANOVA in comparison to the control group.

Studies have demonstrated that plasma treatment, when applied at appropriate intensity and duration, enhances the proliferation of skin cells and related cell types [33]. However, excessive treatment intensity or prolonged duration, specifically beyond a threshold of 50 s, inhibits the proliferation of these cells [34]. To ascertain the optimal duration for plasma treatment, we established gradients of treatment durations at 0 s, 15 s, 30 s, and 45 s, maintaining consistent culture conditions and ensuring that treatment durations did not exceed 50 s (Figure 3A). Following the culture period, cell viability assays indicated that the results increased with longer plasma treatment durations, with the 30 s treatment group achieving the peak rate (Figure 3B). Beyond 30 s, cell viability exhibited a dose-dependent decline; nevertheless, the 45 s treatment group still demonstrated higher viability than the 0 s experimental group (Figure 3B). To further investigate the effect of plasma treatment on cell maturation, we conducted Hoechst staining at the conclusion of the culture period to assess the proportion of first polar body formation. The results indicated that a notable proportion of cells across four experimental groups exhibited signs of nuclear maturation (Figure 3A). Quantitative analysis revealed that the 30-s treatment group had the highest maturation rate (Figure 3C). Prior to 30 s, the maturation rate increased with longer treatment durations; a decline in maturation rate was observed beyond 30 s, although the 45 s treatment group still outperformed the control group (Figure 3C). These experimental findings suggested that LTP promotes both the sharp growth of cells and the maturation of oocytes within the in vitro primary culture system, establishing 30 s as the optimal treatment duration for oocytes.

### 3.4. Effect of LTP on Extracellular Active Substances

**Figure 4:**
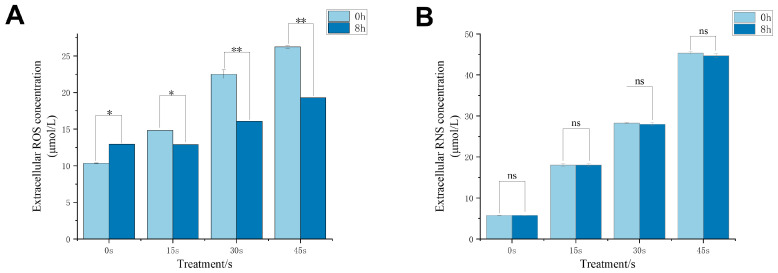
Changes in extracellular bioactive substances after LTP treatment. (**A**) Changes in extracellular ROS concentration at 0 h and 8 h. (**B**) Changes in NO concentration at 0 h and 8 h. The findings are presented as the mean ± standard deviation (SD) obtained from three distinct experiments. For the conclusion, we used ANOVA to evaluate statistical significance, where * *p* < 0.05 and ** *p* < 0.01 indicate comparisons to the control group.

Earlier studies have established that hydrogen peroxide is one of the most crucial reactive oxygen species (ROS) generated by LTP, and nitric oxide (NO) is also one of the most important [35]. As the duration of LTP treatment was prolonged, the levels of NO and hydrogen peroxide in the culture medium were elevated gradually (Figure 4). NO levels after LTP treatment in all experimental groups increased in oocyte primary culture, but they did not show marked changes in content after 8 h; that is, the levels were very similar (Figure 4B). On the contrary, the levels of ROS in all experimental groups after 8 h of in vitro primary culture were lower than the levels that were recorded in all groups immediately after LTP treatment (Figure 4A). The experimental results showed that the redox environment of the culture system had undergone a transient change after LTP treatment. Combined with the results of intracellular ROS, GSH, and NAC, this indicated that the effect of LTP on oocytes occurred simultaneously with the alteration of redox-related reactions.

### 3.5. Effects of LTP on Intracellular Oxidative Stress in Oocytes

**Figure 5:**
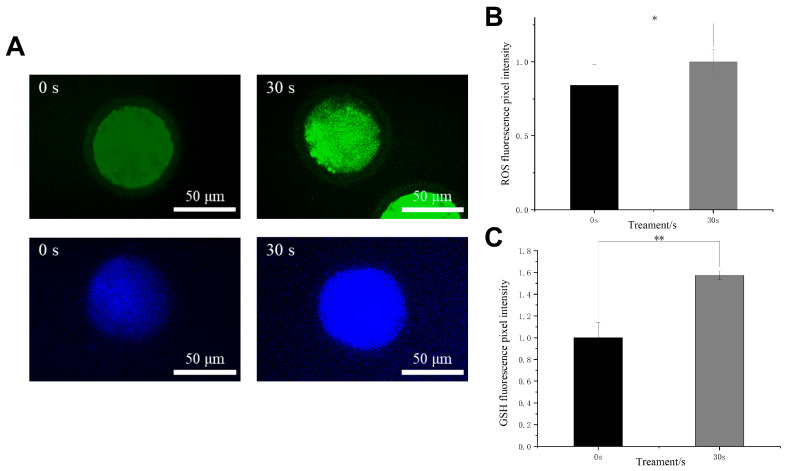
The effect of LTP treatment on the antioxidant capacity of porcine oocytes. (**A**). Representative fluorescence images depicting intracellular ROS and GSH levels for both the 0 s control group and the 30 s LTP-treated group. The scale bar measures 50 µm. (**B**). Comparison of relative intracellular ROS levels between the experimental group and the control group. (**C**). Comparison of relative intracellular GSH levels between the experimental group and the control group. These conclusions are expressed as the mean ± standard deviation (SD) from three unconnected experiments. Statistical significance was assessed through ANOVA, with * *p* < 0.05 and ** *p* < 0.01 indicating a comparison to the control group.

The level of intracellular oxidative stress correlates closely with the concentration of intracellular ROS and GSH, and they have also been known as a critical indicator for evaluating intracellular antioxidant capacity. In this study, through ROS and GSH intracellular staining, LTP treatment elevated both intracellular reactive oxygen species and glutathione levels in the treated group (Figure 5), consistent with the previously observed trend in extracellular ROS dynamics. This observation is consistent with the notion that plasma-induced changes in the extracellular environment may influence intracellular redox balance. The Western blot results showed a marked elevation in the expression levels of the antioxidant transcription factor Nrf2 in porcine oocytes after LTP treatment, in contrast to the control group (Section 3.7). These results indicated that LTP treatment was accompanied by intracellular redox-related alterations. Previous studies have shown that modulation of oxidative stress and mitochondrial status during oocyte IVM can be associated with improved maturation and developmental competence [36]. These changes may reflect either an adaptive redox response or a compensatory antioxidant response to LTP-induced oxidative stress. Therefore, these results supported an association between LTP treatment, altered intracellular redox status, and maturation-related outcomes.

### 3.6. Effect of LTP on Mitochondrial Membrane Potential

**Figure 6:**
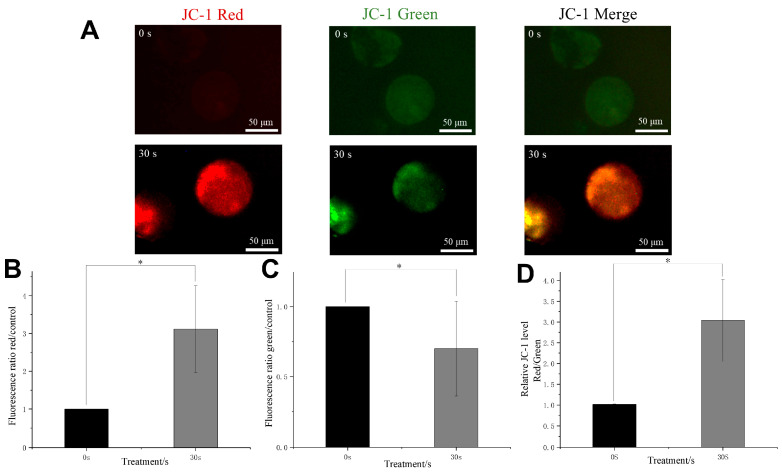
The effect of LTP on mitochondrial membrane potential in oocytes. (**A**) Representative high magnification fluorescence images illustrating the red and green mitochondrial membrane potential in oocytes subjected to LTP for 30 s, in comparison to the control group at 0 s. Scale bar = 50 µm. (**B**,**C**) The intensities of red and green fluorescence in both the LTP-treated and control groups. (**D**) The ratio of red fluorescence to green fluorescence intensity in the group treated for 30 s and the control group. Data are expressed as mean ± standard deviation (SD) from three independent experiments. The statistical significance was analyzed using ANOVA, with * *p* < 0.05.

Oocytes contain a substantial number of mitochondria, which serve as critical indicators for assessing oocyte quality and developmental potential [37]. Mitochondrial dysfunction leads to the production of ROS, triggering oxidative stress that adversely affects oocyte quality [38]. In this study, we utilized JC-1 dye to stain cultured oocytes for the evaluation of their mitochondrial membrane potential (MMP). JC-1 accumulates in cells based on their mitochondrial potential; cells with intact mitochondrial potential convert JC-1 into aggregates, emitting red fluorescence (590 nm), while cells with diminished or lost mitochondrial potential exhibit green fluorescence (530 nm) in the form of monomers. As illustrated in Figure 6A–C, LTP treatment induced an enhancement in mitochondrial membrane potential within cells, evidenced by a decrease in green fluorescence and an increase in red fluorescence. Using quantitative methods, we observed that the ratio of red to green fluorescence in oocytes treated with LTP was markedly greater than that observed in the control group, suggesting an increased level of MMP in the experimental group (Figure 6D). The experimental results indicate that during the in vitro primary culture phase, LTP treatment was accompanied by alterations in intracellular redox-related status and mitochondrial membrane potential (MMP). The GSH-related changes and elevated MMP coincided with maturation-associated outcomes, suggesting that LTP treatment may be associated with changes in the cytoplasmic functional state of oocytes, along with reduced apoptotic signaling and enhanced expression of maturation-related proteins (Section 3.8). These coordinated changes are consistent with a more favorable cytoplasmic functional state; however, the possibility of stress-related mitochondrial hyperpolarization cannot be excluded.

### 3.7. Effect of LTP on Signaling Pathways in Oocytes

**Figure 7:**
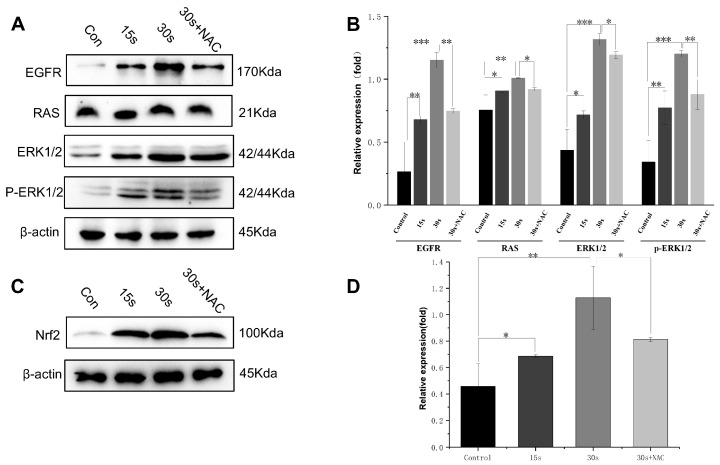
The Nrf2 protein along with proteins related to the EGFR/ERK signaling pathway in oocytes treated using LTP. (**A**,**B**) The quantitatively measured expression levels of relevant proteins across various experimental groups using WB analysis. (**C**,**D**) The expression of Nrf2 in the different groups using WB analysis. *β*-actin served as the loading control. Results are presented as mean ± standard deviation (SD) from three independent experiments. ANOVA was utilized to determine statistical significance, where * *p* < 0.05, ** *p* < 0.01, and *** *p* < 0.001 denote comparisons to the control group.

Previous research has shown that the activation of EGFR and ERK1/2 triggers the initiation of meiosis in oocytes, which enhances the efficiency of in vitro culture and subsequent developmental competence [39]. Furthermore, relative research has indicated that EGFR activation influences the expression of the Nrf2 protein, which plays a crucial role in regulating cell proliferation [40]. To explore whether LTP treatment was accompanied by changes in the expression of EGFR/ERK-related proteins and Nrf2, as well as its correlation with oocyte maturation-related responses, we performed a series of experiments. The findings indicate that LTP treatment was associated with increased expression of EGFR, ERK1/2, and p-ERK1/2, whereas RAS expression showed only modest variation across the experimental conditions, and it also enhanced the expression of the Nrf2 protein (Figure 7A,C). Notably, within 30 s of LTP application, a time-dependent enhancement was demonstrated (Figure 7B,D). These results suggested that LTP treatment was associated with alterations in EGFR/ERK-related proteins and an upregulation of Nrf2 expression, indicating that redox-sensitive signaling pathways might be involved in the maturation-related responses observed in primary oocytes cultured in vitro. Meanwhile, the ROS scavenger NAC attenuated the LTP-induced upregulation of EGFR and downstream signaling proteins (Figure 7B,D). While NAC is commonly used as a modulator of intracellular redox status, it is important to note that it may exert broader biological effects beyond direct ROS scavenging. Therefore, the observed attenuation in the presence of NAC does not constitute definitive evidence of a strictly ROS-dependent mechanism but rather suggests that redox-related processes are involved in mediating LTP-induced signaling activation. Integrating previous research findings with the results of the present study, the alterations in EGFR/ERK-related signaling were likely not isolated events, but rather closely associated with intracellular redox-related status changes [41,42].

### 3.8. LTP Modulates Maturation- and Apoptosis-Related Proteins

**Figure 8:**
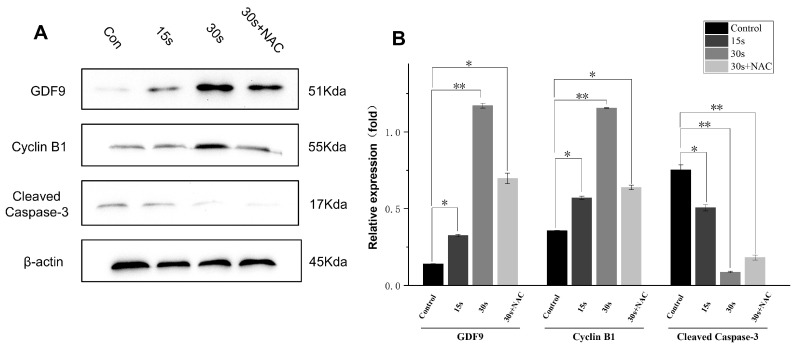
GDF9, Cyclin B1, and cleaved Caspase-3 protein in oocytes treated by LTP. (**A**) Western blot results of GDF9, Cyclin B1, and cleaved Caspase-3 protein. (**B**) The quantitatively measured expression levels of relevant proteins across various groups. Results are presented as mean ± standard deviation (SD) from three independent experiments. ANOVA was utilized to determine statistical significance, where * *p* < 0.05 and ** *p* < 0.01.

To provide molecular evidence beyond morphological observations, we further analyzed the expression of GDF9, Cyclin B1, and cleaved Caspase-3 (Figure 8). GDF9 and Cyclin B1 expression increased progressively from the 0 s to the 30 s group and decreased in the 30 s + NAC group, whereas cleaved Caspase-3 showed the opposite trend. These results indicated that LTP at 30 s was associated with enhanced expression of maturation-related proteins and reduced apoptotic activity. Therefore, the interpretation of LTP-induced maturation was supported by molecular evidence. These molecular changes indicate that the optimal LTP condition is associated with a maturation-supportive and less apoptosis-prone cellular state. Importantly, these findings provide more direct biological evidence.

## 4. Discussion

In this study, we established an in vitro primary culture system for oocytes and demonstrated that LTP enhanced both the survival and maturation rates of DOs by enhancing the growth rate of oocytes and promoting relevant morphological changes [1,43]. Since in the present study, we employed immature oocytes isolated from preantral follicles, whose in vitro culture encompasses both the growth phase and the terminal nuclear maturation stage, LTP-treated oocytes also exhibited increases in oocyte diameter, first polar body extrusion, and the proportion of oocytes with normal perivitelline space morphology, suggesting more favorable growth- and maturation-associated morphological features during culture [3]. According to Figure 2, the proportion of oocytes exhibiting normal perivitelline space morphology in the LTP experimental group, no matter the LTP treatment time or method, was greater than that observed in the control group, further confirming the promoting effect of LTP [4,5]. These changes were accompanied by altered redox-related parameters in the culture system. Under the optimal condition (30 s), LTP induced transient redox-related changes that may be associated with the observed maturation-associated responses (Figure 5); however, the present data does not establish a definitive ROS-triggered adaptive antioxidant mechanism. In contrast, excessive exposure may lead to elevated oxidative stress, which partially offsets these beneficial effects; thus, the biological outcome of LTP is determined by a balance between ROS-mediated signaling and oxidative damage. The enhanced resistance of oocytes to oxidative stress, along with the increased expression of the antioxidant-related protein Nrf2 in the experimental group, also supports this hypothesis [44,45]. Furthermore, oocytes in the LTP-treated group showed a higher mitochondrial membrane potential (Figure 6), suggesting altered mitochondrial functional status during culture. This conclusion aligns with previous studies on LTP-promoted cell proliferation, where LTP also exerted regulatory effects on cellular metabolism [46,47,48]. Under the optimal LTP condition, the coordinated increase in GDF9 and Cyclin B1 and the reduction in cleaved Caspase-3 are consistent with a more maturation-supportive and less apoptosis-prone cellular state. These molecular changes provide additional biological support beyond morphology alone, but they do not by themselves distinguish beneficial adaptation from redox-related stress responses. Moreover, the effects of LTP were dependent on treatment duration and frequency, with the 30 s and LTP (6×) conditions showing the most favorable outcomes among those tested. Overall, these findings suggest that appropriately controlled LTP exposure is associated with improved maturation-related outcomes in cultured oocytes, while the underlying mechanism remains to be further clarified [49].

The EGFR/ERK signaling pathway plays a pivotal role in porcine oocyte growth, maturation, and the bidirectional communication between oocytes and surrounding cumulus cells by regulating key cellular processes [50]. EGFR, positioned upstream in this signaling cascade, functions as a primary signal receptor. Upon ligand binding, EGFR undergoes autophosphorylation, initiating the activation of RAS and subsequently triggering the downstream signaling cascade. ERK, a critical downstream effector, is primarily responsible for modulating oocyte growth, differentiation, and maturation [51]. Previous studies demonstrated that the activation of EGFR on cumulus cells further led to stimulation of oocyte EGFR/ERK signaling [52], and this activation caused meiosis resumption in oocytes, oocyte maturation, and cumulus cell expansion; earlier study findings align with those of the present study in that the activation of the EGFR/ERK signaling pathway eventually enhances oocyte quality [53,54,55]. Notably, the expression profiles of key proteins involved in this pathway observed in earlier studies also align with our current findings. According to all these results, our results suggest that LTP treatment was associated with changes in EGFR/ERK-related proteins, and the upregulation of Nrf2 expression, suggesting that these two components may cooperatively contribute to LTP-related oocyte maturation responses [56,57]. In the same culture, the changes in survival rates and first polar extrusion rates in the LTP-treated group were found to be closely related to the observed changes in protein expression; those findings indicated that LTP treatment assists in the early survival and maturation of oocytes [56]. The attenuation of LTP-induced signaling and maturation-related protein expression by NAC suggests a potential involvement of redox regulation in this process, which was crucial for promoting oocyte development; this mechanism is consistent with those of other studies on LTP-promoted fibroblast cell proliferation [58]. These moderate levels of ROS produced by LTP play crucial roles in stimulating key signaling pathways that boost cellular stressed resistance and development. These results highlight that LTP was a beneficial treatment to ameliorate oocyte quality in assisted reproductive technologies, warranting further exploration of its underlying molecular mechanism. Because the primary objective of this research was to evaluate the effects of LTP treatment on the survival, growth, and maturation of denuded oocytes during in vitro primary culture, rather than on post-fertilization development, fertilization and embryo development assays were not carried out in the present study, but the improvements in mitochondrial membrane potential, intracellular redox balance, and Nrf2 expression all provide supportive molecular context. The lack of direct assessment of medium physicochemical changes (e.g., pH or osmolarity) and cell damage markers represents a limitation of this study.

In comparison with other studies, LTP technology offered significant and novel advantages for improving the growth and multiplication of oocytes. Unlike methods using cumulus cell co-culture, the LTP treatment considerably reduced the risk of cell contamination and phenotypic variation [59,60]. Likewise, the LTP mechanism of action is different from that studied by adding external hormones to the culture medium [61]. This independence reduced the chances of improper hormone doses and was more in line with the way things develop on their own. As a result, this independence increased safety and practicality for experiments [62,63]. While methods based on adding small-molecular-weight substances such as antioxidants or growth factors have been reasonably well-studied [64,65], DOs under these culture conditions are devoid of protection against microbial and oxidative stress and are less resistant. The sterilization effect of LTP [66] made it possible to rapidly reduce the microbial load in the culture environment while transiently altering the medium by generating reactive oxygen species, which optimized the redox signaling microenvironment around the cells and effectively prevented cell damage caused by the adaptive accumulation of reactive oxygen species [67].

## 5. Conclusions

In summary, this study was the first to introduce LTP technology into an in vitro primary culture system of denuded oocytes derived from preantral follicles in the ovarian cortex. The optimal processing parameters for LTP treatment using flexible plasma pads were determined through different durations of LTP treatment. This study demonstrated that LTP treatment improved survival and maturation-related outcomes in porcine denuded oocytes in vitro culture conditions. These effects were accompanied by redox-related changes, altered mitochondrial membrane potential, changes in EGFR/ERK-related proteins, increased Nrf2 expression, and more favorable maturation- and apoptosis-related molecular profiles. NAC attenuated part of these LTP-associated changes, supporting the possible involvement of redox-related regulation, although a strictly ROS-dependent mechanism was not established. This study provides new technical insights and theoretical foundations for improving the efficiency of the in vitro primary culture of oocytes.

## Data Availability

The original contributions presented in this study are included in the article. Further inquiries can be directed at the corresponding author.

## Abbreviations

The following abbreviations are used in this manuscript:

LTP	Low-temperature plasma
DOs	Denuded oocytes
PVS	Perivitelline space
NAC	N-acetyl-L-cysteine
ROS	Reactive oxygen species
RNS	Reactive nitrogen species
GSH	Glutathione
NO	Nitric oxide
MTT	3-(4,5-dimethylthiazol-2-yl)-2,5-diphenyl tetrazolium bromide
MMP	Mitochondrial membrane potential
Nrf2	Nuclear factor erythroid 2–related factor 2
EGFR	Epidermal growth factor receptor
ERK	Extracellular signal-regulated kinase
P-ERK	Phosphorylated extracellular signal-regulated kinase
RAS	Rat sarcoma
